# Cosmetic Medicine and *Aesthetic Surgery Journal*: The Beautiful Bond Between Noninvasive Aesthetic Medicine and Plastic Surgery

**DOI:** 10.1093/asj/sjag014

**Published:** 2026-04-07

**Authors:** Julius W Few, Sachin M Shridharani

Welcome to the special Cosmetic Medicine issue of *Aesthetic Surgery Journal* (*ASJ*), in celebration of the journal's 30th anniversary. In this editorial, we describe the evolution and innovation of nonsurgical and minimally invasive aesthetic medicine and how this focus of the specialty continues to mold plastic surgery at large. For 3 years in a row, the Cosmetic Medicine section of *ASJ* has been the highest-volume subject section with regard to manuscript submissions (Figure 1). This growth speaks volumes about how far nonsurgical cosmetic medicine has come in recent decades, as well as the growth of *ASJ* and the aesthetic plastic surgery specialty.

**Figure 1.**
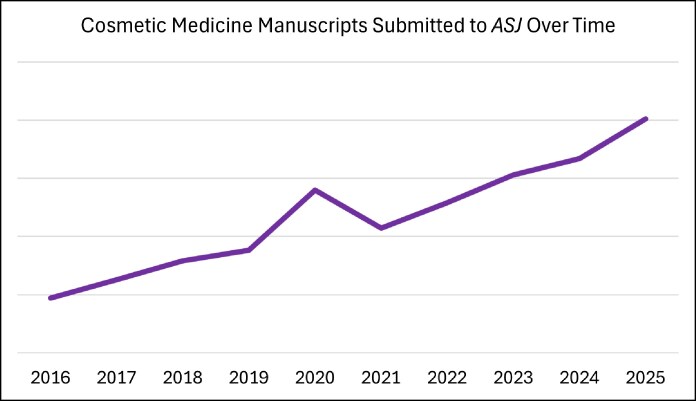
Line graph illustrating the rate of increase of peer-reviewed manuscripts submitted to the Cosmetic Medicine section of *Aesthetic Surgery Journal* over a 10-year period.

## COSMETIC MEDICINE AND AESTHETIC SURGERY: A BRIEF HISTORY

The field of noninvasive and nonsurgical cosmetic medicine has evolved greatly in the years since the launch of *ASJ* 3 decades ago. In 1992, Dr Jean and Alastair Carruthers published their results of the injection of botulinum neurotoxin type A (BTX-A) for the treatment of glabellar lines, a groundbreaking development for this specialty.^1^  *ASJ* quickly took up the mantle in publishing on the subject of injectable neuromodulators, even in the earlier iterations of this publication, back when it was a quarterly news publication, *Aesthetic Surgery Quarterly,* a smaller-scale publication written for members of The American Society for Aesthetic Plastic Surgery.^2^ In 1996, this quarterly newsletter grew into *ASJ*, a scholarly publication with rigorous peer review, and thus the new, revolutionized era of *ASJ* was born. On the topic of injectables, thinking back to the days of the mid and late 1990s when the idea of fillers meant doing 2 separate skin testing sessions (6 weeks apart) for collagen, then injecting it with hope that it would last 3 to 6 months for a lip enhancement, the scale of advancement within our field is astounding. Back in those days, many (arguably most) patients suffered horrible pain from these injections, and the “dental block” was the only way to inject lips in those days. To think that, in the early days of doing BTX-A injections (Botox; Allergan, Irvine, CA), young plastic surgeons were often told by more senior plastic surgeons that “squirting” Botox into someone's face would never replace a “good old-fashion facelift!” Many felt that Botox was a simple fad, and one that would disappear soon. What was once labeled as a “fad” now dominates as the world's most frequently performed aesthetic procedure.^3^ Following BTX-A injections, dermal fillers take the penultimate spot in popularity, with millions of treatments per year. Aesthetic plastic surgeons at large now fully appreciate how critical it is to be facile, not only with the scalpel, but also with the syringe. Today, we can inject products that erase wrinkles, volumize deficient fat pads, stimulate collagen, destroy adipocytes, and dissolve dermis tethering collagen septae. Many would argue that the innovation in cosmetic medicine occurs at a rate outpacing traditional surgical procedures.^4^

**Figure 2.**
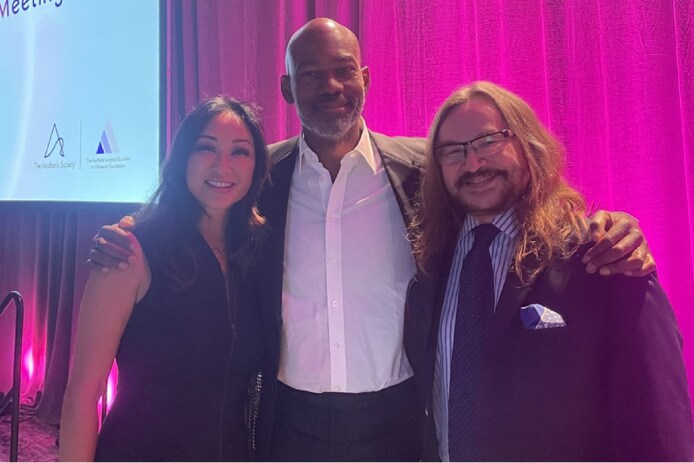
Dr Julius Few (middle) photographed with his respected mentor, Dr Michael Kane (right), and Dr Jackie Yee (left) at The Aesthetic Society's annual meeting in Miami, FL in 2023.

For those who saw its potential, the realization was clear that nonsurgical cosmetic medicine was more than a fad—it was a path to the future. Thanks to the mentorship and pioneering spirit of Dr Michael Kane, who, in the early days of its use, was the largest single user of Botox in the world, the research side of cosmetic medicine came to the fore (Figure 2). It was very clear to those who were plugged into this world that aesthetic medicine could 1 day transcend plastic surgery or dermatology. To that end, it was imperative that plastic surgery had a seat at the table and worked closely with thought leaders in dermatology, oculoplastic surgery, and facial plastic surgery to help create this new specialty. Thankfully, Mark Jewell, Foad Nahai, Robert Singer, Renato Saltz felt the same and helped Dr Julius Few to convince others in plastic surgery of this imperative. Dr Jewell, as president of The American Society for Aesthetic Plastic Surgery (now The Aesthetic Society) at the time, formed the injectable safety coalition and named Dr Few, the ambassador for plastic surgery, helping to put aside disagreements between specialties and work with one another to make sure the “core 4” would help to protect patients' interests and safety with an ever-growing injectable space.

## COSMETIC MEDICINE AND *AESTHETIC SURGERY JOURNAL*

**Figure 3.**
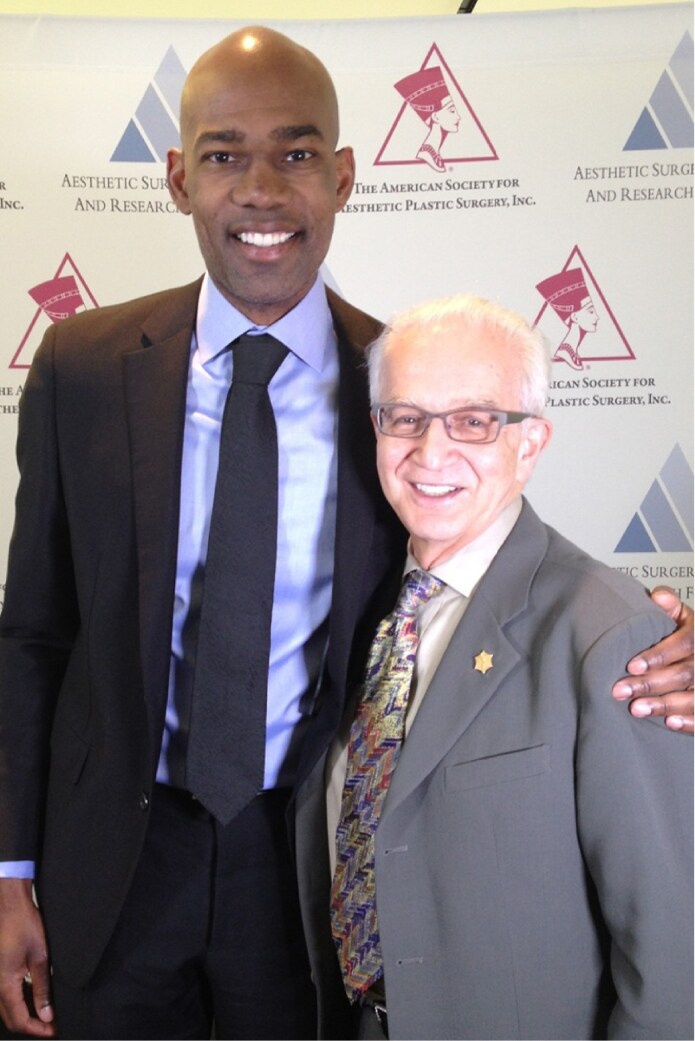
Dr Julius Few (left) and Dr Foad Nahai (right) photographed in April 2014 at The Aesthetic Society's annual meeting in San Francisco, CA.

**Figure 4.**
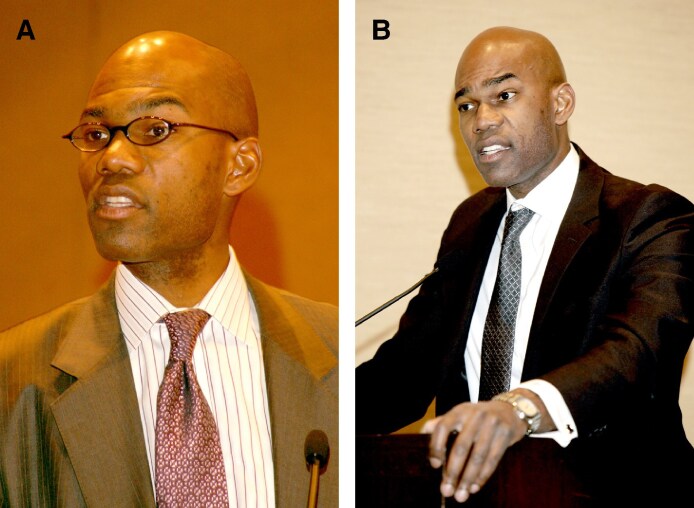
Dr Julius Few presenting at (A) The Aesthetic Society's annual meeting in San Diego, CA in 2008 and (B) again in San Francisco, CA in 2014.

**Figure 5.**
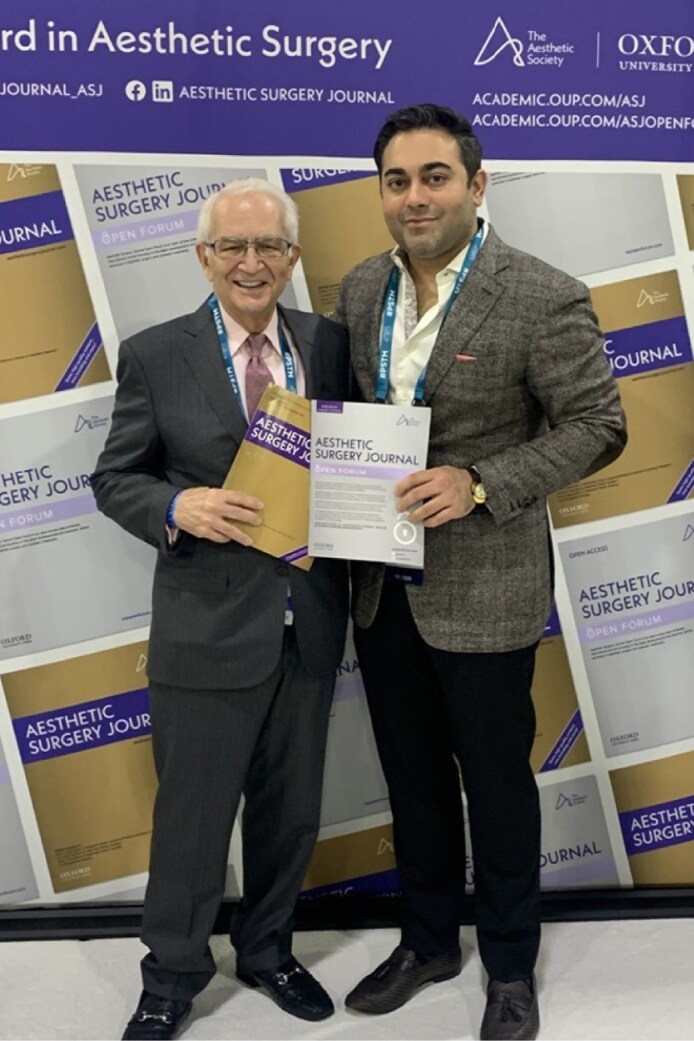
Dr Foad Nahai (left) and Dr Sachin Shridharani (right) photographed together at The Aesthetic Society's annual meeting in Miami, FL in 2023.

**Figure 6.**
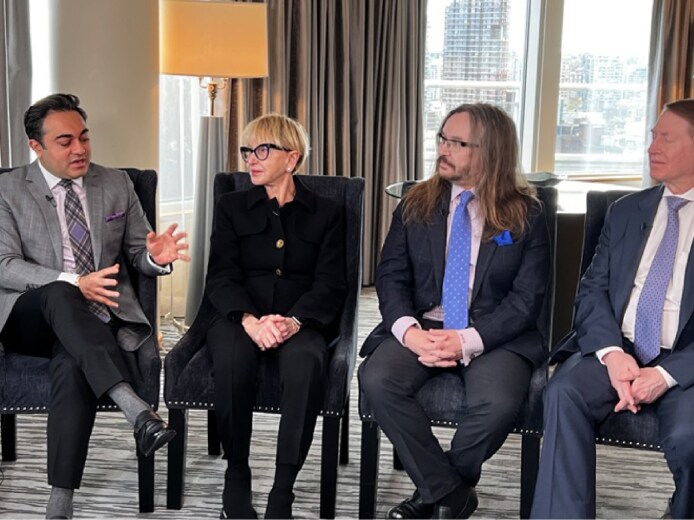
Dr Sachin Shridharani (far left) moderates a 2024 video roundtable titled “Innovations in Injectables: An Expert Video Roundtable Discussion” with (left to right) Dr Jean Carruthers, Dr Michael Kane, and Dr Lawrence Bass as commentators, as published in **ASJ* Open Forum*.^6^.

**Figure 7.**
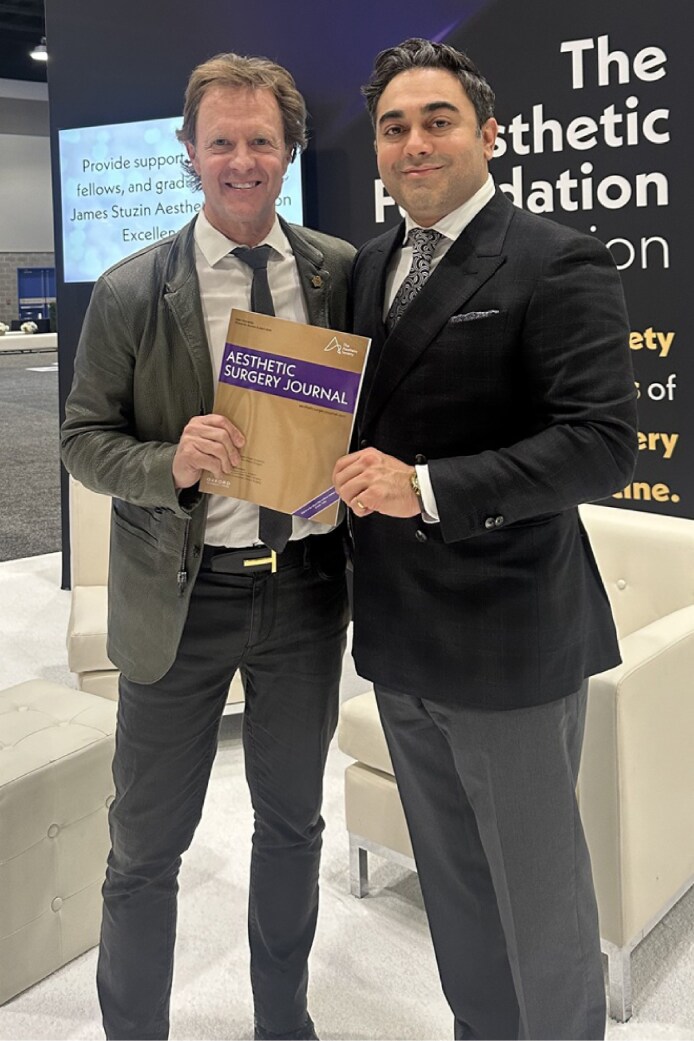
Dr Jeffrey Kenkel (left) and Dr Sachin Shridharani (right) photographed together at The Aesthetic Society's annual meeting, The Aesthetic MEET, in Vancouver, Canada in 2024.

This effort, harnessing cosmetic medicine in plastic surgery and beyond, is a vital legacy. There was no way to definitively predict how momentous and impactful this effort would grow to be, now larger in consumption per year than cosmetic surgery by several folds.^5^ The idea that there would be a discipline that could help lift sagging tissue, erase unwanted fat, and eradicate sun damage with little downtime and minimal pain (all without surgically excision skin) seemed more akin to an episode of Star Trek, not plastic surgery, in the year 2000. It is not a wonder that the individuals named above were all past presidents of The Aesthetic Society and were revolutionary in their thinking and artistry. A special shout-out is because of Foad Nahai for having the foresight and wisdom to formally launch Cosmetic Medicine in 2009 as a dedicated section of *ASJ* under his keen editorship and leadership (Figures 3, 4). Not only did he truly put *ASJ* “on the map” for all cosmetic specialties, but he also saw the true potential for this subspecialty in plastic surgery. He entrusted this section first to Dr Few, and eventually to Dr Sachin Shridharani as a co-section editor in 2022, and it has been a great honor to help the section grow (Figures 5-7).

*ASJ* has progressed immensely during its 30-year lifetime, with a surging impact factor to match. It is a source of scientific innovation in which to take great pride, and there is no question why it is one of the most-cited sources for cosmetic medicine in dermatology, facial plastics, and oculoplastic surgery. Included here are a number of influential, widely read, and highly cited articles spanning the last several decades.^7-34^ These important papers illustrate that with growing adoption of a given application, there is a growing need for innovation in treatment and prevention of complications. Anyone performing and/or overseeing individuals performing nonsurgical cosmetic medicine should read all of these articles, because they are truly gold-standard references. These papers are not only of historical relevance but also serve as a reminder of the groundwork upon which great aesthetic clinical practice has been built, such as Claudio de Lorenzi's 2-part landmark article series on fillers, “Complications of Injectable Fillers,” which are 2 of the most highly cited and highest read papers in the history of *ASJ*.^7,8^ These pivotal papers helped Dr Few in particular to save major morbidity for a beautiful young female patient who was injected with filler to her face by an untrained nurse working out of her home.

The development and refinement of filler management tools is essential, but so is the inclusion of important research tools in plastic surgery. For example, the publication of several personal investigator-initiated studies and modifications of the FACE-Q principle, such as “Patient Perceived Benefit in Facial Aesthetic Procedures: FACE-Q as a Tool to Study Botulinum Toxin Injection Outcomes,” published in *ASJ* in 2016.^9^ From the clinical application of low-level light therapy, to how sleep distorts facial aging, to the impact of video conferencing on appearance dissatisfaction, to the management and prevention of filler complications and beyond, these highly cited and impactful *ASJ* publications in the Cosmetic Medicine section offer our readers vital, current, data-driven material in their pursuit of aesthetic excellence and patient satisfaction.^10-20^ Not one to rest on laurels, *ASJ* is continually publishing new research on the patient safety, treatment efficacy, expansion, and innovation of injectable neuromodulators and dermal fillers for aesthetic treatment.^21-31^

The primary charge of scholarly research on cosmetic medicine is to encourage the researcher, the intellectually curious, to address a current topic of importance in treating the nonsurgical candidate, a call to action that inspired Dr Few and several colleagues to look at long-term (3-year) satisfaction with absorbable suture suspension of the face—facial thread lifting.^32^ Currently, over the past year, Dr Few has begun experimenting with the use of nonsurgical tissue tightening, using focused ultrasound and needle radiofrequency as a tool to maintain postfacelift efficacy after 2 years or more. This is based on the extensive work in the area for primary use.^33,34^ The advent of these techniques and technologies for aesthetic maintenance is exciting and, one might argue, analogous to maintaining a new car or home. Cosmetic medicine has allowed the creative nature of aesthetic surgery to blossom into a much more creative reality, much like to innovation of technicolor going to 4 or 8 K definition.

## PREDICTIONS FOR THE FUTURE

By the time *ASJ* turns 40, 10 years from now, artificial intelligence will almost certainly have a role in all forms of aesthetic plastic surgery and nonsurgical innovation. It will no longer be “how do we get there?” but rather “where do we want to go?.” An abiding hope is that the holy grail, addressing atrophic or senile skin changes, will be largely reversible with composite principles in the not-too-distant future. The practice of aesthetic surgery will become much more predictable because of a more algorithmic approach support by technology and predictive artificial intelligence models, and the birth of truly undetectable cosmetic treatments, if the patient wishes, is on the horizon. In addition, the genesis of truly preventative strategies for outward signs of aging, similar to what is done with systemic inflammation prevention and overall physical health, is surely within our reach. What if there are genetic markers that can predict one's tendency to form early steatoblepharon, lower eyelid bags? Could we use radiofrequency or microfocused ultrasound devices to preemptively prevent it, or some future technology to abate the issue altogether? Will we, as a society, stop chasing 1 beauty ideal and focus on individual beauty and realistic, but ideal, outward appearance that is age ideal? Will we see a true integration between health, wellness, and beauty? Certainly the next decade and beyond will have viable answers to these questions, to the betterment of our society and in the pursuit of the optimal path of aging.

### Personal Predictions: Julius Few, MD

In my lifetime, I would really like to see technology that either predicts and/or offers a solution to early recurrent neck banding, postfacelifting. Because someone who mainly does facial cosmetic surgery, while fortunately not frequently, the return of neck banding is the bane of my professional existence. If this could go away forever, along with patients wanting only natural results, what a true pleasure or nirvana facelifting would be in my world. This would allow less-aggressive neck procedures and the advent of truly elegant, nondetectable plastic surgery, something that I have aspired to for many years.

### Personal Predictions: Sachin Shridharani, MD

In my lifetime, I would like to see continued evolution of technologies and treatments that allow for the restoration of skin integrity. True large-volume elastogenesis could be the holy grail for maintenance of skin quality, reduction/elimination of stretch marks, and afford us optimization of excisional surgical procedures both above and below the clavicle. As we learn in surgical training, “you can’t stop the clock” and this applies to the aging process. This often-unwanted maturation process spares no one, ergo treatments restoring skin resiliency are an unmet need and will change the landscape of Cosmetic Medicine.

## CONCLUSIONS

In conclusion, we are as co-editors of the Cosmetic Medicine section are proud stewards of *ASJ*. Dr Jeffrey Kenkel has successfully transitioned from Associate Editor to Editor-in-Chief and has shown us all that he intends to take the vision of *ASJ* championed by Dr Foad Nahai (and Drs Stanley Klatsky and Robert Bernard before him) to its next level. Jeff has the vision and instinct to truly build the journal to even greater heights, and we are honored to call him a friend and colleague. This journal represents more than a source for research and innovation, it represents a constant reminder to pursue your professional truth and hold one's clinical practice to the highest standard of betterment for our patient experience. The addition of Dr Sachin Shrindharani as a co-section editor has been a full circle moment, given our first meeting when he was a medical student. We hope that our shared drive to pursue excellence will serve the journal and our specialty well. We are moved by and grateful for those before us, who paved the way for us to contribute to the continuing legacy of plastic surgery.
